# Impact of *TSC2* loss on progression-free survival in uterine carcinosarcoma: A retrospective analysis

**DOI:** 10.20407/fmj.2025-001

**Published:** 2025-08-06

**Authors:** Ryoko Ichikawa, Tamotsu Sudo, Kyohei Takada, Akiko Ohwaki, Mayuko Ito, Yusuke Shimizu, Mayu Takeda, Eiji Sugihara, Tetsuya Takimoto, Haruki Nishizawa

**Affiliations:** 1 Department of Obstetrics and Gynecology, Fujita Health University, School of Medicine, Toyoake, Aichi, Japan; 2 Department of Cancer Genetics & Genomics, Fujita Health University, School of Medicine, Toyoake, Aichi, Japan; 3 Faculty of Health and Medical Sciences, Aichi Syukutoku University, Nagakute, Aichi, Japan; 4 Division of Gene Regulation, Oncology Innovation Center, Fujita Health University, Toyoake, Aichi, Japan; 5 Research Promotion Headquarters, Open Facility Center, Fujita Health University, Toyoake, Aichi, Japan; 6 Division of Date Science, Oncology Innovation Center, Fujita Health University, Toyoake, Aichi, Japan

**Keywords:** Uterine carcinosarcoma, *TSC2* loss, mTOR inhibitor

## Abstract

**Objective:**

Uterine carcinosarcoma (UCS) is a rare and aggressive gynecological cancer with high recurrence rates and is associated with a poor prognosis. It is also characterized by a high frequency of copy number alterations (CNAs). This study aimed to determine which gene CNA contributes to progression-free survival (PFS) in patients with UCS to identify potential prognostic biomarkers and therapeutic targets.

**Methods:**

DNA was extracted from formalin-fixed paraffin-embedded tissues of surgical specimens from 24 patients with UCS who were treated at Fujita Health University. Using the PleSSision-Rapid test, the mutation information of 145 cancer genes was analyzed. Oncoplot analysis was used to visualize gene mutation profiles. χ^2^ test, Kaplan–Meier analysis, and log-rank test were used for statistical analyses.

**Results:**

The most frequently observed gene mutation was *TP53* in the 24 UCS cases studied, while genes associated with the PI3K/AKT/mTOR signaling pathway, including *PIK3CA*, *PTEN*, *PIK3R1*, and *PIK3R2* were commonly detected. In the χ^2^ test analysis, *TP53* loss (*p*=0.029), *PIK3CA* amplification (*p*=0.034), and *TSC2* loss (*p*=0.034) were significantly associated with recurrence. Kaplan–Meier survival analysis demonstrated a significant association between *PIK3CA* amplification and *TSC2* loss with PFS, as determined by the log-rank test (*p*<0.05).

**Conclusion:**

In patients with UCS, *TSC2* loss is linked to poorer PFS, highlighting its utility as a prognostic marker. The association between *TSC2* loss and increased recurrence risk highlights the potential therapeutic advantage of targeting the mTOR pathway in *TSC2*-deficient tumors.

## Introduction

Uterine carcinosarcoma (UCS), also referred to as malignant mixed Müllerian tumor, is a rare and highly aggressive uterine malignancy that accounts for approximately 3%–5% of all uterine cancers but disproportionately contributes to uterine cancer-related mortality.^1–4^ UCS presents unique challenges in its diagnosis and treatment, characterized by a biphasic histological composition of both epithelial and mesenchymal components.^5,6^ Early-stage UCS, encompassing stages I and II, has significantly poorer prognoses and survival outcomes than other high-risk uterine cancers, including clear cell carcinoma, serous carcinoma, and grade 3 endometrioid adenocarcinoma.^7–9^

UCS exhibits a unique genomic landscape that is prominently characterized by a high frequency of copy number alterations (CNAs)^10,11^. CNAs, involving gains or losses of large genomic regions, are implicated in UCS tumorigenesis and progression, reflecting the genomic instability that is a hallmark of this cancer type. These molecular markers are not only valuable for prognostication but also hold promise as potential targets for therapeutic intervention. Therapies specifically targeting these genetic drivers could provide a novel approach to treating UCS, with the potential to enhance survival outcomes in patients with unfavorable prognostic indicators.

Tuberous sclerosis complex 2 (*TSC2)* is a critical tumor suppressor gene that encodes the protein tuberin, which, together with the *TSC1*-encoded protein hamartin, forms the TSC complex.^12,13^ This complex is a central regulator of the mechanistic target of rapamycin complex 1 (mTORC1) pathway, a vital signaling cascade that controls cell growth, proliferation, and metabolism in response to nutrient availability, energy levels, and cellular stress. By acting as a GTPase-activating protein for the Rheb protein, the TSC complex inhibits mTORC1, thereby limiting mTOR activity.^12^ *TSC2* inactivation results in sustained mTORC1 activation, leading to unregulated cell growth and proliferation, a hallmark of tumorigenesis. *TSC2* mutations or deletions are associated with numerous cancers and have been identified as critical drivers in tumor types with heightened mTOR pathway activity. Loss of *TSC2* function has been particularly implicated in cancers that demonstrate aggressive clinical behavior, including renal cell carcinoma and certain brain tumors.^14,15^ Due to its central role in mTORC1 regulation, *TSC2* has garnered attention as a potential therapeutic target.^16–19^ In cancers with *TSC2* loss, targeted therapies using mTOR inhibitors such as everolimus and temsirolimus have demonstrated promise in preclinical and clinical studies, providing a strategy to counteract the growth-promoting effects of mTOR hyperactivation. Studies on the TSC/mTOR pathway highlight its critical role in tumor biology, positioning *TSC2* as a crucial target for developing precision medicine strategies to treat cancers associated with mTOR dysregulation.

This study aimed to explore the genetic landscape of UCS, with a focus on identifying CNAs that influence prognosis. Through the examination of these factors in relation to progression-free survival (PFS), this research seeks to inform strategies for precision medicine in UCS, providing guidance for the development of therapies that target the specific molecular mechanisms underlying its aggressive behavior.

## Methods

### Study Design and Patient Selection

The Institutional Review Board at Fujita Health University approved this retrospective cohort study (HM24-205 and HM23-251), adhering to the ethical guidelines for human research. Clinical records of UCS cases diagnosed between 2013 and 2023 were reviewed, restricting the sample set to cases with confirmed UCS diagnoses, adequate tissue samples for genetic analysis, and comprehensive follow-up data.

The inclusion criteria were histologically confirmed UCS, archived tissue suitable for genetic testing, and clinical outcome data, specifically PFS. Exclusion criteria were as follows: incomplete records, inadequate tissue samples, or a documented history of other concurrent malignancies. Ultimately, 24 patients satisfied the eligibility criteria and were considered the study cohort.

### Data Collection

Demographics, clinical characteristics, and treatment history data were extracted from the electronic medical records, including age at diagnosis, tumor stage, treatment type, and follow-up information. Two independent pathologists specializing in gynecologic oncology verified the pathological findings, such as histological subtype and tumor grade. Clinical data also included recurrence status, allowing the examination of correlations between genetic findings and recurrence risk.

### Genetic Analysis

DNA from all tumor samples was sequenced in-house using the PleSSision-Rapid-Neo testing platform, with slight modifications to established methods.^20^ Enriched libraries were then sequenced using the paired end (150 bp×2) sequencing method with the NextSeq2000 NGS system (Illumina, San Diego, CA, USA).^20^ Sequence data were analyzed as previously described using the GenomeJack bioinformatics pipeline (Mitsubishi Electric Software Corporation; http://genomejack.net/). A list of detectable cancer-related genes and genetic rearrangements based on this genomic analysis is provided in Supplementary Table 1.

### Statistical Analysis

Categorical variables were compared using the χ^2^ test. Survival analyses were conducted using Kaplan–Meier survival curves, and differences between groups were assessed with the log-rank test. All analyses were conducted using SPSS version 22 with statistical significance set at *p*<0.05.

## Results

### Patients’ Characteristics

**Figure 1:**
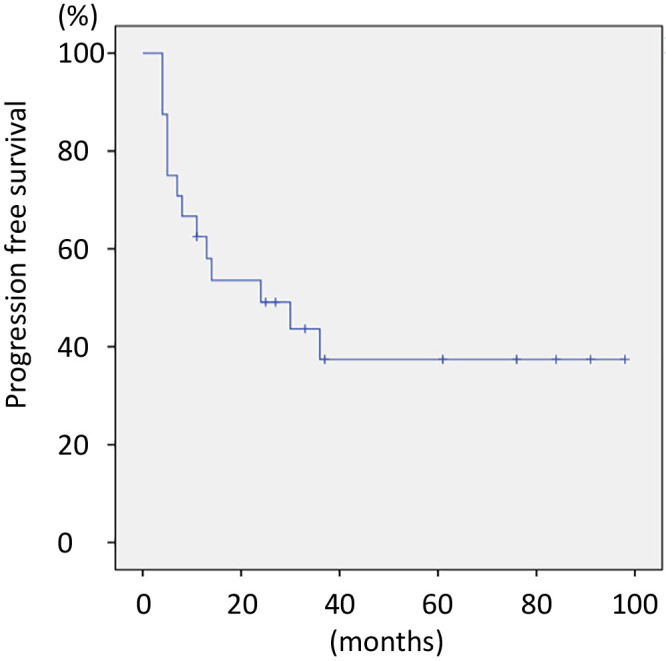
Progression-free survival (PFS) curves for all cases of uterine carcinosarcoma. Kaplan–Meier curve demonstrating the PFS of patients with uterine carcinosarcoma (n=24). Median PFS was 24 months, as determined from the survival curve.

Table 1 illustrates the clinical characteristics of the 24 patients with UCS included in the study. The median age at diagnosis was 68 years (range, 51–92) and all patients were postmenopausal. Of the 24 patients, 71% were classified with stage I UCS, which differs from the typical clinical presentation where UCS is often detected at an advanced stage.^1,3^ This represents a distinctive aspect of the current study. Histopathologically, all UCS cases showed biphasic features consisting of both sarcomatous and carcinomatous components. Endometrioid carcinoma was the most prevalent (46%) among the carcinomatous components, followed by serous carcinoma (38%). The sarcomatous components included 67% heterologous and 33% homologous types. The PFS curves for all cases of UCS are shown in Figure 1. The median PFS was 24 months, with most recurrences occurring within this period. Patients who remained recurrence-free beyond 24 months often attained long-term PFS, with some cases revealing no progression beyond 100 months. These findings demonstrate the importance of monitoring during the first 2 years after treatment while revealing the potential for durable disease control in patients without early recurrence. The recurrence rates were evaluated based on stage, carcinoma component, and sarcoma component (Table 2). Recurrence rates were significantly associated with disease stage (*p*=0.008). All stage III and IV patients experienced recurrence, whereas 70% of stage I patients remained recurrence-free. Regarding the carcinoma component, recurrence rates failed to differ significantly between serous (six recurrence cases vs. three non-recurrence cases) and non-serous subtypes (eight recurrence cases vs. seven non-recurrence cases; *p*=0.521). Similarly, no significant difference was observed in the recurrence rates between heterologous (11 recurrence cases vs. 5 non-recurrence cases) and homologous sarcoma components (3 recurrence cases vs. 5 non-recurrence cases; *p*=0.143).

### Mutation Status in UCS

**Figure 2:**
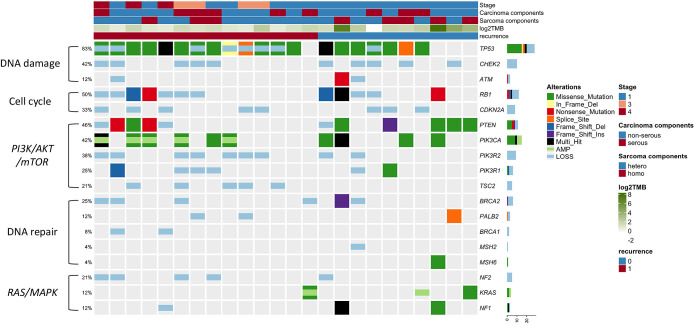
Oncoplot demonstrating pathological mutations and copy number alterations in uterine carcinosarcoma. Genes are grouped by function, including DNA damage, cell cycle regulation, PI3K/AKT/mTOR pathway, DNA repair, and RAS/MAPK signaling. Furthermore, tumor mutational burden and recurrence status are depicted. Two categories of genomic alterations—somatic mutations (e.g., missense, nonsense, or frameshift variants) and copy number alterations (e.g., amplifications or deletions)—are concurrently displayed. In instances where both a somatic mutation and a copy number alteration are present in the same gene within a single sample, the alterations are superimposed within the same cell of the plot. The percentages indicate the proportion of cases with alterations among the 24 samples analyzed.

Among 24 patients with UCS, 75 gene alterations with a mutational prevalence of 4% or higher were found (Supplementary Table 2). The most commonly altered gene was *TP53* (83%), followed by *STK11* (54%) and *RB1* (50%). Analyses revealed that the tumors not only harbored mutations characteristic of sarcoma, such as *RB1* (50%), *ATRX* (38%), and *FBXW7* (17%) mutations, but also possessed mutations commonly associated with endometrial adenocarcinoma, including *PTEN* (42%), *PIK3CA* (42%), and *AKT* (17%) mutations. These findings are consistent with previously reported data on UCS, indicating that, on a molecular level, UCS possesses characteristics of both sarcoma and adenocarcinoma.^10^ We constructed an oncoprint focusing on pivotal genes involved in UCS-related carcinogenesis to examine the genetic landscape of UCS, including DNA damage response, cell cycle regulation, PI3K/AKT/mTOR signaling pathway, DNA repair mechanisms, and the RAS/MAPK signaling axis (Figure 2). With the high CNA prevalence in UCS, tumor suppressor genes *CHEK2* (42%), *PIK3R2* (38%), *CDKN2A* (33%), and *TSC2* (21%) were found to exhibit only gene deletions, with no single nucleotide variants detected. A detailed examination of genes associated with DNA repair was conducted, highlighting those involved in homologous recombination—such as *BRCA1*, *BRCA2*, and *PALB2*—and genes related to mismatch repair, including *MLH1*, *MSH2*, and *MSH6*. Three loss-of-function mutations were found in the *BRCA2*, *PALB2*, and *MSH6* genes among the variants identified (Supplementary Table 3).

### Relationship Between CNAs and Recurrence

UCS is characterized by a high prevalence of CNAs,^10^ which prompted us to explore the relationship between CNAs and recurrence. The associations between CNAs in specific genes and recurrence status were analyzed (Table 3). Our analysis demonstrated significant associations among several genes. Within the PI3K/AKT/mTOR pathway, *PIK3CA* amplification (*p*=0.034) and *TSC2* loss (*p*=0.034) were significantly more frequent in cases with recurrence. In the DNA damage pathway, *TP53* loss was also significantly associated with recurrence (*p*=0.040). No significant associations were identified for CNAs in the RAS/MAPK pathway, DNA repair pathway, or among cell cycle regulators.

### PFS Analysis

**Figure 3:**
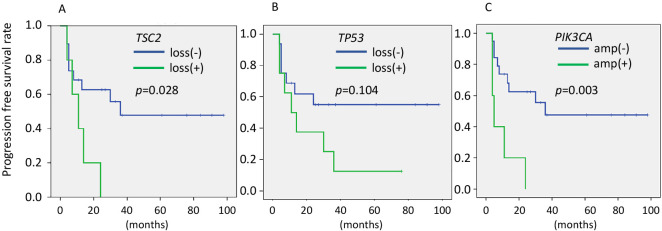
Kaplan–Meier curves of PFS by copy number alteration (CNA) status. (A) PFS curves stratified by *TSC2* loss status. (B) PFS curves stratified by *TP53* loss status. (C) PFS curves stratified by *PIK3CA* amplification status. Statistical analyses were conducted using the log-rank test.

Kaplan–Meier survival curves for PFS, illustrated in Figure 3, showed significant differences in outcomes between patients with and without *TP53* loss, *PIK3CA* amplification, and *TSC2* loss. Median PFS was shorter in those with *TSC2* loss (*p*=0.028) and *PIK3CA* amplification (*p*=0.003), underscoring the prognostic relevance of *TSC2* and *PIK3CA* CNAs in UCS.

## Discussion

In this cohort of 24 patients with UCS, median PFS was 24 months, with most recurrences occurring within this critical timeframe. Patients who remained recurrence-free beyond 24 months frequently achieved long-term PFS, underscoring the importance of vigilant monitoring during the first 2 years post-treatment. In this window, early detection of recurrence allows for timely medical interventions that may improve clinical outcomes. This study aimed to identify molecular factors associated with recurrence to improve prognostic stratification and enhance clinical management.

*TP53* mutations, observed in 83% of cases, are consistent with prior research highlighting their prevalence in UCS.^21^ However, in this cohort, significant association was not observed between *TP53* mutations and recurrence (data not shown). Conversely, *TP53* loss was significantly associated with recurrence, indicating its potential role in tumor progression. This finding supports the hypothesis that biallelic inactivation of *TP53*, through mutation and allelic loss, contributes to the risk of recurrence. Although the sample size of this study was limited, these results corroborate with the established role of *TP53* as a critical tumor suppressor and warrant further investigation in larger, multi-institutional cohorts to validate this association and explore underlying mechanisms.

One of the most significant findings of this study is the association between *TSC2* loss and reduced PFS, representing a novel contribution to the understanding of UCS. *TSC2* plays a critical role as a negative regulator of the mTOR pathway, primarily through the suppression of mTOR complex 1 (mTORC1) activity, thereby maintaining controlled cell growth and proliferation. The loss of *TSC2* disrupts this pathway, potentially leading to unregulated mTOR activation and subsequent tumor progression.^12,13^ Additionally, our results indicate that *PIK3CA* gene amplification is significantly associated with recurrence, a finding consistent with those of previous studies.^22,23^ *PIK3CA* gene amplification was detected only in tumors that concomitantly harbored *PIK3CA* mutations (data not shown), suggesting a synergistic effect leading to enhanced *PIK3CA* activation. This dual alteration may serve as a molecular driver of tumor progression and could, at least in part, account for the aggressive clinical behavior and poor prognosis observed in UCS patients.^22^ Holst et al. have demonstrated that *PIK3CA* amplification is a strong prognostic marker in several cancers. Interestingly, their work also highlighted that *PIK3CA* amplification does not inherently correlate with the activation of downstream signaling pathways, including *AKT* and mTOR, indicating that these pathways can function independently of canonical pathway activation.^23^ Conversely, our data provide evidence that *TSC2* loss may directly contribute to mTOR activation, suggesting a potential mechanistic explanation distinct from the *PIK3CA*-driven pathway. This distinction underscores the complexity of mTOR pathway regulation in UCS and indicates that *TSC2* and *PIK3CA* may exert their effects on tumor biology via divergent mechanisms.

Furthermore, the activation of the mTOR pathway due to *TSC2* loss highlights its therapeutic potential. mTOR inhibitors could offer a targeted treatment approach for UCS patients with *TSC2* alterations. Identification of such patients may allow personalized treatment strategies, addressing the unmet need for effective therapies in UCS management.

In conclusion, this study identifies loss of *TSC2* as a novel prognostic marker and potential therapeutic target of UCS. Integrating molecular biomarkers such as *TSC2* into clinical practice could enhance risk stratification, guide therapeutic decision-making, and ultimately enhance patient outcomes. Through the advancement of our understanding of UCS biology, these findings represent a meaningful step toward personalized management of this aggressive malignancy.

## Figures and Tables

**Table 1:** Clinical and pathological characteristics of 24 uterine carcinosarcoma cases

Characteristics	Number of cases (%)
Median age (range)	68 (51–92)
Menopausal status	
– Pre-menopausal	0 (0)
– Post-menopausal	24 (100)
Stage	
– I	17 (71)
– II	0 (0)
– III	4 (16)
– IV	3 (13)
Sarcoma component	
– Homologous	8 (33)
– Heterologous	16 (67)
Carcinoma component	
– Serous	9 (38)
– Endometrioid	11 (46)
– Other	4 (16)

**Table 2:** Recurrence outcomes categorized by stage, sarcoma component, and carcinoma component in 24 cases of uterine carcinosarcoma. Comparisons include Stage I versus Stage III+IV, as well as variations in sarcoma and carcinoma components. Statistical analyses were performed using the χ^2^ test.

Clinical findings	number of recurrence cases	number of no recurrence cases	*p* value
Stage			
– I	7	10	0.008
– II	0	0	
– III	4	0	
– IV	3	0	
Sarcoma component			
– Heterologous	11	5	0.143
– Homologous	3	5	
Carcinoma component			
– Serous	6	3	0.521
– Non-serous	8	7	

**Table 3:** Relationship between copy number alterations (CNA) and recurrence in uterine carcinosarcoma. The table compares recurrence and non-recurrence cases based on CNAs (amplification, loss, or none) in genes grouped by functional pathways. Statistical analyses were performed using the χ^2^ test.

Pathway/Functional Category	gene	CNA	number of recurrence cases	number of no recurrence cases	*p* value
PI3K/AKT/mTOR	*PIK3CA*	amp	5	0	0.034
		none	9	10	
	*PIK3R1*	loss	3	1	0.459
		none	11	9	
	*PIK3R2*	loss	7	2	0.134
		none	7	8	
	*PTEN*	loss	2	1	0.754
		none	12	9	
	*TSC2*	loss	5	0	0.034
		none	9	10	
RAS/MAPK	*KRAS*	amp	1	1	0.803
		none	13	9	
	*NF1*	loss	1	0	0.388
		none	13	10	
	*NF2*	loss	4	1	0.239
		none	10	9	
DNA damage	*TP53*	loss	7	1	0.040
		none	7	9	
	*CHEK2*	loss	5	5	0.484
		none	9	5	
	*ATM*	loss	1	1	0.803
		none	13	9	
DNA repair	*BRCA1*	loss	2	0	0.212
		none	12	10	
	*BRCA2*	loss	4	1	0.459
		none	10	9	
	*PALB2*	loss	2	0	0.212
		none	12	10	
	*MSH2*	loss	0	1	0.227
		none	14	9	
cell cycle	*RB1*	loss	5	2	0.404
		none	9	8	
	*CDKN2A*	loss	5	3	0.770
		none	9	7	
