# Supplementary material for: Impact of TSC2 loss on progression-free survival in uterine carcinosarcoma: A retrospective analysis
Source: Fujita Med J. 2025 Aug 6;11(4):155–60. doi: 10.20407/fmj.2025-001 (PMC12576405; doi:10.20407/fmj.2025-001)
Supplement: Supplementary file 1 — Supplementary Tables [file fmj-11-155-s001.pdf]

Supplementary Table 1

Plession Rapid gene list

ABL1	*ACTN4*	*AKT1*	*AKT2*	*AKT3*	*ALK*	*APC*	*AR*
ARAF	*ARID1A*	*ARID2*	*ASXL1*	*ATM*	*ATRX*	*AXIN1*	*AXL*
BAP1	*BARD1*	*BCL2L11*	*BRAF*	*BRCA1*	*BRCA2*	*BRIP1*	*CARD11*
CCND1	*CD274*	*CD79B*	*CDH1*	*CDK12*	*CDK4*	*CDKN2A*	*CHEK2*
CREBBP	*CRKL*	*CSF1R*	*CTNNB1*	*CUL3*	*DDR2*	*DNMT3A*	*ECT2L*
EGFR	*ENO1*	*EP300*	*EPCAM*	*ERBB2*	*ERBB3*	*ERBB4*	*ESR1*
EZH2	*FANCA*	*FANCD2*	*FANCE*	*FBXW7*	*FGFR1*	*FGFR2*	*FGFR3*
FGFR4	*FH*	*FLT3*	*GNA11*	*GNAQ*	*GNAS*	*GRIN2A*	*HRAS*
IDH1	*IDH2*	*IGF1R*	*IGF2*	*IL7R*	*JAK1*	*JAK2*	*JAK3*
KDM6A	*KDR*	*KEAP1*	*KIT*	*KMT2D*	*KRAS*	*MAP2K1*	*MAP2K2*
MAP2K4	*MAP3K1*	*MAP3K4*	*MDM2*	*MDM4*	*MED12*	*MET*	*MLH1*
MSH2	*MSH6*	*MTOR*	*MYC*	*MYCN*	*MYD88*	*NF1*	*NF2*
NFE2L2	*NOTCH1*	*NOTCH2*	*NOTCH3*	*NPM1*	*NRAS*	*NRG1*	*NT5C2*
NTRK1	*NTRK2*	*NTRK3*	*PALB2*	*PBRM1*	*PDGFRA*	*PDGFRB*	*PIK3CA*
PIK3RI	*PIK3R2*	*PMS2*	*POLD1*	*POLE*	*PRKCI*	*PTCH1*	*PTEN*
RAC1	*RAC2*	*RAD51C*	*RAF1*	*RB1*	*RET*	*RHOA*	*ROS1*
SETBP1	*SETD2*	*SMAD4*	*SMARCA4*	*SMARCB1*	*SMO*	*SPOP*	*SRC*
STAT3	*STK11*	*TERT*	*TP53*	*TSC1*	*TSC2*	*VHL*	*WT1*
XPC							

∗ The 11 genes underlined are also targets for fusion gene detection.

Supplementary Table 2

All Altered gene detected in uterine carcinosarcoma

gene	prevalence (%)	gene	prevalence (%)	gene	prevalence (%)
TP53	83	*CDH1*	17	*SMARCB1*	8
STK11	54	*EP300*	17	*SPOP*	8
RB1	50	*FBXW7*	17	*ARID2*	4
CHEK2	42	*GRIN2A*	17	*AXL*	4
PIK3CA	42	*KMT2D*	17	*BCL2L11*	4
PTEN	42	*MAP3K1*	17	*BRIP1*	4
ATRX	38	*PRKCI*	17	*CD274*	4
PIK3R2	38	*ATM*	12	*CTNNB1*	4
CDKN2A	33	*CDK12*	12	*EGFR*	4
SMARCA4	33	*CREBBP*	12	*ENO1*	4
AXIN1	25	*KRAS*	12	*EPCAM*	4
BRCA2	25	*NF1*	12	*ERBB3*	4
FANCA	25	*PALB2*	12	*JAK3*	4
KDM6A	25	*POLE*	12	*KDR*	4
KEAP1	25	*RAD51C*	12	*KIT*	4
MAP2K4	25	*MAP3K1*	8	*MDM4*	4
MAP3K4	25	*BRCA1*	8	*MSH2*	4
MYC	25	*CUL3*	8	*MSH6*	4
PIK3R1	25	*ERBB2*	8	*NT5C2*	4
TSC2	25	*ESR1*	8	*PDGFRA*	4
APC	21	*FGFR1*	8	*POLD1*	4
NF2	21	*GNAS*	8	*PTCH1*	4
SMAD4	21	*PMS2*	8	*TSC1*	4
AKT2	17	*RAF1*	8	*WT1*	4
ARID1A	17	*SETBP1*	8	*XPC*	4

Gene	Nucleotid or Protein change	Variant Allele Frequency	Pathogenicity
BRCA2	p.Asn1784fs	0.26	Pathogenic
PALB2	c.109-2A>C	0.34	Likely pathogenic*
MSH6	c.4001G>A	0.27	Pathogenic

Supplementary Table 3.
Genetic mutations were annotated for pathogenicity using ClinVar and OncoKB^{TM}.
The *PALB2* c.109-2A>G variant has been classified as likely pathogenic in the ClinVar database (Accession: VCV000182778.42), which implicates that the c.109-2A>C variant at the same splicing site may hold a similar evaluation.
